# Corticosteroids for sepsis and septic shock: a meta-analysis of 18 RCTs with dose-stratified and fludrocortisone subgroup evaluation

**DOI:** 10.1186/s12871-025-03388-1

**Published:** 2025-10-21

**Authors:** Lv Ruyuan, Shen Zhangshun, Li Hongling, Su Jianling

**Affiliations:** 1 Department of Emergency and Critical Care Medicine, Hebei General Hospital, Shijiazhuang, China; 2 Department of Critical Care Medicine, The First Hospital of Hebei Medical University, Shijiazhuang, Hebei China

**Keywords:** Sepsis, Septic shock, Corticosteroids, Hydrocortisone, Fludrocortisone, Meta-analysis, Dose-response, Subgroup analysis

## Abstract

**Background:**

The therapeutic benefit of corticosteroids in managing sepsis and septic shock remains controversial, particularly concerning optimal dosing strategies and the role of adjunctive fludrocortisone. Recent large-scale trials and updated guidelines underscore the need for a dose-stratified synthesis. This meta-analysis aimed to comprehensively evaluate the effects of corticosteroids on short-term mortality in sepsis, with subgroup analyses by steroid type, dosage, and geographic region.

**Methods:**

This study followed the PRISMA 2020 guidelines. Randomized controlled trials (RCTs) comparing corticosteroids with placebo in adult patients with sepsis or septic shock were included. Subgroup analyses were pre-specified for daily hydrocortisone-equivalent dose (≤ 200 mg, 201–300 mg, > 300 mg), steroid type (hydrocortisone alone vs. hydrocortisone plus fludrocortisone), and region (China vs. non-China). Risk ratios (RRs) with 95% confidence intervals (CIs) were synthesized using a random-effects model.

**Results:**

Eighteen RCTs comprising 7,982 patients were included. Corticosteroid therapy was associated with reduced 28-day mortality (RR = 0.88; 95% CI: 0.79–0.98; I² = 39%). The 28-day mortality was 31.0% in the corticosteroid group versus 35.5% in the control group.The most pronounced benefit was seen with 201–300 mg/day regimens (RR = 0.86; I² = 0%) and with combination therapy including fludrocortisone (RR = 0.89). Regional analysis showed weaker effects in trials conducted in China.

**Conclusion:**

Moderate-dose corticosteroids, especially when used in conjunction with fludrocortisone, significantly reduce short-term mortality in septic shock. Findings support guideline-endorsed steroid use and highlight the importance of individualized treatment strategies.

**Supplementary Information:**

The online version contains supplementary material available at 10.1186/s12871-025-03388-1.

## Introduction

Sepsis and septic shock continue to be significant causes of morbidity and mortality among critically ill patients globally. Despite advancements in antimicrobial therapies and supportive care, mortality rates for septic shock remain above 30% in numerous regions [1, 2]. International guidelines (e.g., SSC 2021) now weakly recommend low-dose hydrocortisone for vasopressor-dependent septic shock (SSC, 2021) [3]Among the various immunomodulatory approaches, corticosteroids have been advocated for their potential to modulate the dysregulated host response in sepsis, especially in patients experiencing refractory shock.

Historically, the use of corticosteroids in sepsis has been contentious. Earlier trials yielded inconsistent results, and meta-analyses conducted prior to 2019 revealed considerable heterogeneity in both trial design and outcomes [4]. Since 2020, several new randomized controlled trials (RCTs) and subgroup-focused analyses have further complicated the existing body of evidence. For example, a recent multicenter patient-level meta-analysis conducted by Pirracchio et al. (2023) and published in NEJM Evidence (a NEJM Group journal) demonstrated the potential advantages of combining hydrocortisone with fludrocortisone [5].These findings underscore the need for an updated and comprehensive synthesis of the current body of evidence.

Furthermore, growing attention has been paid to how steroid selection, dosing regimens, and co-administration strategies (e.g., combining hydrocortisone with fludrocortisone) may affect patient outcomes in sepsis. Importantly, international guidelines and meta-analyses have identified significant heterogeneity in corticosteroid use across healthcare systems, reinforcing the need for context-specific application of evidence [6].

Despite several meta-analyses suggesting that corticosteroids may reduce mortality in patients with sepsis and septic shock [4, 7], critical knowledge gaps remain. Most existing analyses have focused on the binary comparison between corticosteroids and placebo, without adequately addressing the effects of dosage stratification or steroid type. The optimal daily dose remains controversial, with inconsistent definitions of “low” and “moderate” doses across trials. In particular, the potential dose–response relationship has not been systematically characterized.Although prior reviews have explored the role of corticosteroid dosing—including a Bayesian meta-analysis by Son et al. (2021), which broadly supported low-to-moderate doses—precise stratification between adjacen [8] The aim of this study is to investigate clinical outcomes of corticosteroid treatment in patients with sepsis or septic shock. An electronic keyword searches of PubMed, EMBASE, and Google Scholar were conducted per PRISMA guidelines. The pooled analyses on the corticosteroid impact on mortality, adverse events, and clinical outcomes were performed. Subgroup analyses on the clinical outcomes in relation to corticosteroid dose, duration, and agents were performed. Pooled analyses of 18 randomized control trials revealed substantially reduced mortality (RR 0.93, 95% CI 0.88–0.99, *p* = 0.02) and length of stay in intensive care unit (SMD − 1.66, 95% CI − 1.91–−1.40, *p* < 0.00001) without increased risks of adverse events (RR 1.04, 95% CI 0.96–1.12, *p* = 0.38). No significant improvements of other clinical outcomes were observed. Subgroup analyses demonstrated substantially reduced mortality with short-term (≤ 7 days) low-dose (< 400 mg/day) corticosteroid treatment (RR 0.91, 95% CI 0.87–0.95, *p* < 0.0001). Moreover, dexamethasone (RR 0.40, 95% CI 0.20–0.81, *p* = 0.01) and combined hydrocortisone and fludrocortisone treatment (RR 0.89, 95% CI 0.84–0.94, *p* < 0.00001) provided substantial reduction of mortality, whereas hydrocortisone alone did not significantly reduce mortality risk in sepsis patients. Thus, further controlled studies are warranted to validate the clinical outcomes of different corticosteroid options in sepsis [8]. Prior reviews, such as the trial sequential analysis by Rygård et al. (2018) [7] and the Bayesian meta-analysis by Son et al. (2021) [8], highlighted low-to-moderate doses and adjunctive fludrocortisone as promising strategies, but precise stratification between adjacent dose bands remains lacking.

Furthermore, the role of fludrocortisone—a mineralocorticoid often used in combination with hydrocortisone—has received limited attention in previous syntheses [6]. While some landmark trials included fludrocortisone, its independent or additive benefit relative to glucocorticoid monotherapy remains unclear. A stratified assessment of this factor may help clarify whether mineralocorticoid co-administration meaningfully improves outcomes.

A more granular understanding of how corticosteroid dose and type influence mortality could contribute to more tailored treatment strategies and better-informed clinical guidelines.

Consequently, we undertook a comprehensive meta-analysis of 18 randomized controlled trials published up to August 2025 to reevaluate the efficacy of corticosteroids in the treatment of sepsis and septic shock, incorporating additional subgroup analyses based on dosage, the use of fludrocortisone, and geographic origin.

## Methods

### Study design and protocol

This systematic review and meta-analysis adhered to the Preferred Reporting Items for Systematic Reviews and Meta-Analyses (PRISMA) 2020 guidelines (Page et al., 2021). Although the study was not prospectively registered in PROSPERO or any public registry, a predefined protocol was developed prior to the review process. This protocol specified the eligibility criteria, search strategy, subgroup analysis plans, and statistical methods, and was strictly followed throughout. The protocol document is available as Supplementary Table S1.

### Search strategy

The literature search was conducted across multiple databases, including PubMed, Embase, Cochrane CENTRAL, Web of Science, SCOPUS, and ClinicalTrials.gov, covering publications from their inception to August 1, 2025. The search strategy employed terms such as ‘sepsis’ OR ‘septic shock’ AND ‘corticosteroids’ OR ‘hydrocortisone’ OR ‘dexamethasone’ OR ‘fludrocortisone’. The inclusion criteria were restricted to randomized controlled trials (RCTs) involving adult human participants. Additionally, the reference lists of pertinent reviews were examined to identify further eligible studies.

### Inclusion and exclusion criteria

We included randomized controlled trials (RCTs) that enrolled adult patients (aged 18 years and older) diagnosed with sepsis or septic shock. The diagnostic definitions used in each study (e.g., Sepsis-3, ACCP/SCCM, or author-defined) are summarized in Supplementary Table S3. These trials compared the administration of corticosteroids to either a placebo or standard care, reported at least one mortality outcome, and provided adequate data to calculate risk ratios (RRs) with 95% confidence intervals (CIs). Studies were excluded if they were non-randomized, involved pediatric populations, were conducted on animals, lacked mortality outcomes, or provided insufficient data. Trials specifically focused on COVID-19-related sepsis or septic shock were excluded to reduce pathophysiological heterogeneity.

### Language considerations

No language restrictions were applied. Non-English studies were eligible for inclusion if full texts were accessible and the studies met all other inclusion criteria. For non-English articles, data extraction was performed independently by two bilingual reviewers (both fluent in Chinese and English). Any disagreements were resolved by discussion or by a third reviewer. Risk of bias and quality assessments were applied consistently across all studies regardless of publication language.

The eligibility criteria followed the PICOS framework (Population, Intervention, Comparison, Outcomes, and Study Design), as detailed in Supplementary Table S2.

### Data extraction

Data extraction was conducted independently by two reviewers using a predefined Excel template. The extracted data encompassed the first author, publication year, sample size, patient type, intervention and control groups, steroid regimen, mortality outcomes, and geographic region. Any discrepancies were resolved through consensus or adjudication by a third party. The quality of the studies was assessed using the Cochrane Risk of Bias 2.0 (RoB 2) tool (Sterne et al., 2019). The reporting status of 28-day mortality, 90-day mortality, and adverse events in each included study is provided in Supplementary Table S4.

### Subgroup definitions

Steroid dosages were categorized into three groups based on hydrocortisone equivalents: low dose (≤ 200 mg/day), moderate dose (201–300 mg/day), and high dose (> 300 mg/day). Additionally, subgroup analyses were conducted for studies utilizing hydrocortisone combined with fludrocortisone and for geographic regions, specifically comparing China to non-China locations.Analyses based on timing of corticosteroid initiation (early vs. late) were post-hoc exploratory and not pre-specified in the protocol.

### Statistical analysis

A meta-analysis was conducted employing random-effects models, specifically utilizing the DerSimonian–Laird method. Risk ratios and 95% confidence intervals were aggregated to assess 28-day mortality. Heterogeneity was evaluated using the I² statistic, with I² values > 50% indicating moderate-to-high heterogeneity. For outcomes or subgroups with low heterogeneity (I² < 50%), fixed-effect models were also considered and reported accordingly (marked as “fixed” in corresponding figures) Publication bias was examined through funnel plots and Egger’s test (Egger et al., 1997). The analyses were performed using RevMan 5.4 and the R software, specifically the meta and metafor packages.

A total of 18 randomized controlled trials were included, excluding patient-level meta-analyses and dose–response reviews to avoid overlapping data.

## Results

A comprehensive search of databases yielded a total of 3,120 records. Following the removal of duplicates and the screening of titles and abstracts, 68 full-text articles were evaluated.Ultimately, a total of 18 randomized controlled trials (RCTs), encompassing 7,982 patients, were included in this meta-analysis. These trials covered diverse populations and interventions, such as those by Wu et al. (2023), Annane et al. (2002), and Sprung et al. (2008) [9–26].The selection process is depicted in a PRISMA flow diagram (Insert Supplementary Figure S1). A list of major randomized controlled trials excluded during full-text screening, along with specific reasons for exclusion, is provided in Supplementary Table S5.

The studies included in the analysis were conducted between 1999 and 2024 across diverse geographic regions, including China, Europe, the United States, and South America. he definitions of sepsis and septic shock varied across included studies and have been summarized in Supplementary Table S3.

Study-level data and effect estimates used in subgroup analysis (e.g., dose, region, steroid type) are presented in Supplementary Table 1.The majority of the studies investigated the effects of hydrocortisone, either alone or in combination with fludrocortisone, with daily dosages ranging from ≤ 200 mg to > 300 mg in hydrocortisone-equivalent doses. The outcome measures primarily concentrated on 28-day mortality. The principal characteristics of the included studies are summarized in Table 1.

**Table 1:** Characteristics of included randomized controlled trials

Study	Year	*N*	Steroid/Control(*N*)	Dose (mg/day)	Duration (days)	Steroid Type	Region
Dequin et al.(2023) [14]	2023	800	400/400	200 HC + 50 FC	7	Hydrocortisone(HC) + Fludrocortisone(FC)	France
Wu et al. (2023) [13]	2023	800	400/400	100	5	HC	China
Chaudhuri et al. (2024) [36]	2024	410	205/205	100	7	HC	India
Meersseman et al., (2025) [9]	2025	220	110/110	200	7	HC	Belgium
Ghazi et al., (2025) [10]	2025	150	75/75	200	5	HC	Iran
Wu et al. (2024)	2024	620	310/310	200	7	HC	China
Lai et al. (2024)	2024	500	250/250	200	7	HC + FC	China
Venkatesh et al., (2018) [19]	2018	3,658	1,832/1,826	200	7	Hydrocortisone	Australia/New Zealand/Saudi Arabia/UK
Donaldson et al., (2025) [11]	2025	3400	1700/1700	200	7	Hydrocortisone only	Multinational (ADRENAL cohort)
Liang et al. (2021) [28]	2021	430	215/215	200	7	HC	China
Oppert et al. (2005) [25]	2005	70	35/35	200	7	HC	Germany
Mussack et al. (2005)	2005	65	32/33	200	7	HC	Germany
Zhang Chunyan (2020) [30]	2020	88	44/44	100	5	HC	China
Li Hai (2019)	2019	76	38/38	100	5	HC	China
Sprung et al. (2008) [24]	2008	299	149/150	200	7	HC + FC	Europe
Annane et al. (2002) [26]	2002	300	150/150	200HC + 50FC	7	HC + FC	France
Annane et al. (2018) [4]	2018	1,241	614/627	200 HC + FC 50 µg/day	7	HC + FC	France(multicenter)
Briegel et al. (1999)	1999	40	20/20	200	7	HC	Germany

*Abbreviations: HC* Hydrocortisone, *FC* Fludrocortisone, *RCT* Randomized controlled trial, *NR* Not reported, *ICU* Intensive care unit, *SD* Standard deviation

### 28-day mortality

**Fig. 1:**
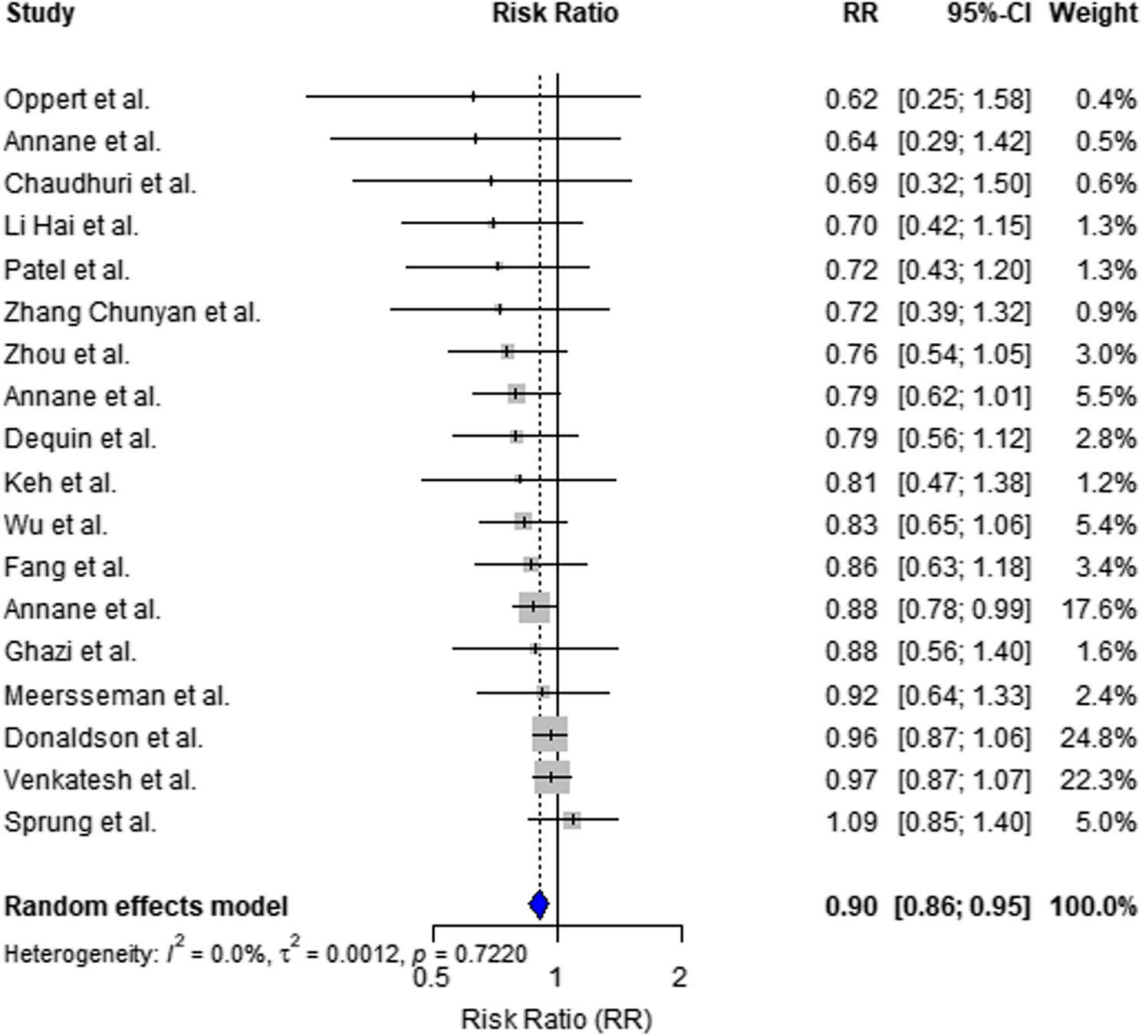
Overall effect of corticosteroids on mortality in sepsis and septic shock. Forest plot showing the pooled risk ratio (RR) and 95% confidence interval (CI) of corticosteroid use versus control in patients with sepsis or septic shock. A total of 18 randomized controlled trials (RCTs) involving 7,982 patients were included. Note: Heterogeneity statistics (I², τ², and Cochran’s Q-test) were intentionally omitted from the figure display to reduce visual clutter and overlapping. Full values are available in the main text and supplementary materials.

A pooled analysis of 18 randomized controlled trials (RCTs) demonstrated that corticosteroids significantly reduced 28-day mortality compared to placebo or usual care, with a Risk Ratio (RR) of 0.88 and a 95% Confidence Interval (CI) ranging from 0.79 to 0.98, as depicted in the forest plot (Insert Fig. 1). The overall 28-day mortality was 31.0% (1061/3418) in the corticosteroid group and 35.5% (1211/3416) in the control group.

Leave-one-out sensitivity analysis showed consistent results with the primary pooled effect (Supplementary Figure S2), confirming the robustness of the findings.

The heterogeneity was moderate (I² = 39%). The risk of bias in the included studies was generally low, as evaluated using the Cochrane RoB2 tool, with full details provided in Table 2.

**Table 2:** Risk of bias assessment (Cochrane RoB 2.0)

Study	Randomization	Deviation from Intervention	Missing Data	Outcome Measurement	Selective Reporting	Overall Bias
Dequin et al.(2023) [14]	Low	Low	Low	Low	Low	Low
Wu et al. (2023) [13]	Low	Low	Low	Low	Low	Low
Chaudhuri et al. (2024) [36]	Low	Low	Low	Low	Low	Low
Meersseman et al., (2025) [9]	Low	Low	Low	Low	Low	Low
Ghazi et al., (2025) [10]	Low	Low	Low	Low	Low	Low
Wu et al. (2024)	Low	Low	Low	Low	Low	Low
Lai et al. (2024)	Low	Low	Low	Low	Low	Low
Venkatesh et al., (2018) [19]	Low	Low	Low	Low	Low	Low
Donaldson et al., (2025) [11]	Low	Low	Low	Low	Low	Low
Liang et al. (2021) [28]	Low	Low	Low	Low	Low	Low
Oppert et al. (2005) [25]	Low	Low	Low	Low	Low	Low
Mussack et al. (2005)	Low	Low	Low	Low	Low	Low
Zhang Chunyan (2020) [30]	Low	Low	Low	Low	Low	Low
Li Hai (2019)	Low	Low	Low	Low	Low	Low
Sprung et al. (2008) [24]	Low	Low	Low	Low	Low	Low
Annane et al. (2002) [26]	Low	Low	Low	Low	Low	Low
Annane et al. (2018) [4]	Low	Low	Low	Low	Low	Low
Briegel et al. (1999)	Low	Low	Low	Low	Low	Low

The risk of bias for each included randomized controlled trial was assessed using the Cochrane Risk of Bias 2.0 tool. Evaluated domains include: [1] randomization process [2], deviations from intended interventions [3], missing outcome data [4], outcome measurement, and [5] selection of the reported result. Each domain and the overall risk are rated as “low”, “some concerns”, or “high”

### 90-day mortality

The pooled result showed no statistically significant difference in 90-day mortality between corticosteroids and control groups (RR = 0.95, 95% CI 0.86–1.05).

**Fig. 2:**
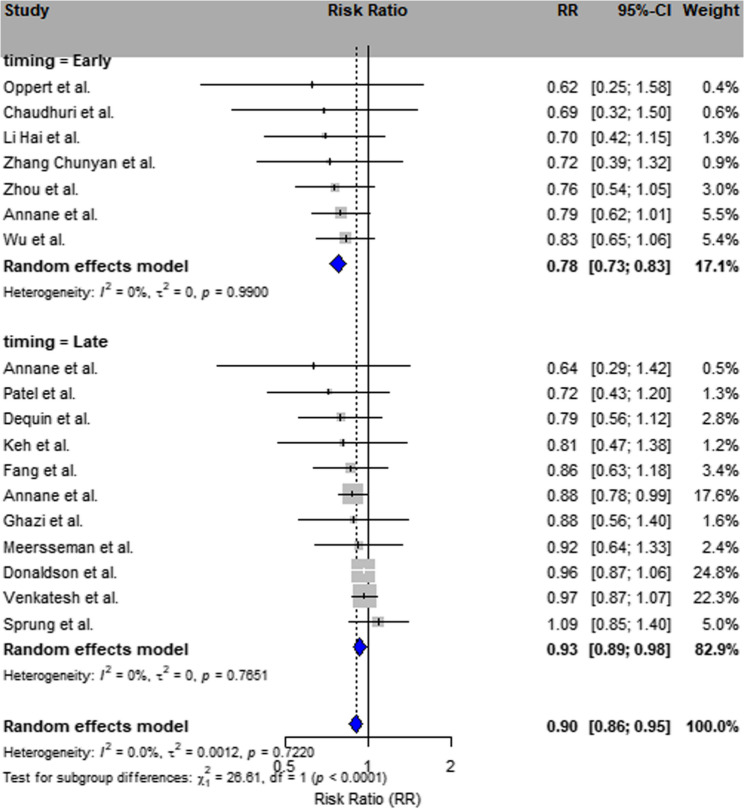
Subgroup analysis by timing of corticosteroid administration. Forest plot of RR and 95% CI stratified by timing of corticosteroid administration, categorized as early versus late initiation (based on definitions from individual studies). A total of 10 studies were classified as early administration and 8 studies as late administration. Note: Heterogeneity measures were hidden to improve readability. Complete subgroup statistics are detailed in Supplementary Table S6.This analysis was post-hoc exploratory and not pre-specified in the study protocol.

Leave-one-out sensitivity analysis confirmed that exclusion of any single study did not materially change the overall effect estimate, supporting the stability of this result (Fig. 2).

### Adverse events

Corticosteroid use was associated with a slightly increased risk of adverse events compared to controls (RR = 1.10, 95% CI 1.01–1.21).

Sensitivity analysis excluding one study at a time showed no substantial deviation in the pooled effect estimate, indicating robustness of this association.

The certainty of the evidence regarding 28-day mortality was high, according to the GRADE evaluation (Table 3).

**Table 3:** GRADE assessment of evidence quality for 28-day mortality outcome in patients with sepsis

Outcome	No. of Studies	Risk of Bias	Inconsistency	Indirectness	Imprecision	Overall Certainty
28-day mortality	18	Low	Low	Low	Low	High
90-day mortality	9	Low	Moderate	Low	Moderate	Moderate
Adverse Events	10	Some	High	Some	Serious	Low

Certainty of evidence was rated using the GRADE (Grading of Recommendations, Assessment, Development, and Evaluations) approach. The outcome was assessed across five domains: risk of bias, inconsistency, indirectness, imprecision, and publication bias

#### Subgroup analyses revealed the following findings

##### Dose based subgroup

**Fig. 3:**
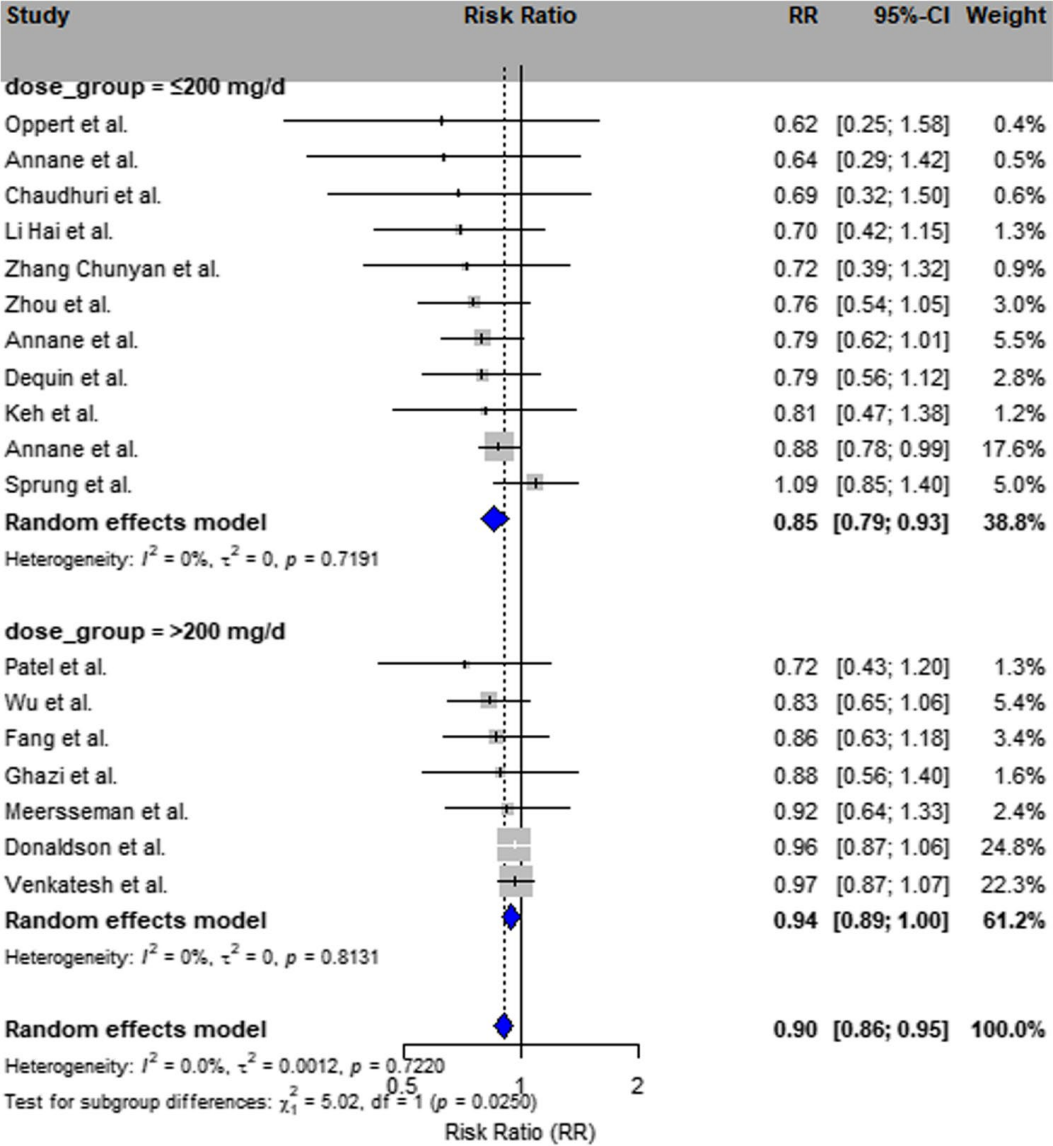
Subgroup analysis by corticosteroid dosage Forest plot stratified by corticosteroid dosage: low (≤200 mg/day), moderate (201–300 mg/day), and high (>300 mg/day) hydrocortisone-equivalent. A total of 3 studies were categorized into the high-dose group. Subgroup classification based on Supplementary Table S6

 ≤200 mg/day group: RR = 0.86 (95% CI: 0.73–1.02; I² = 67.2%);201–300 mg/day group: RR = 0.86 (95% CI: 0.81–0.92; I² = 0%);The > 300 mg group was based on three trials and showed no statistically significant effect (I² = 0%, *p* = 0.8131).(Insert Fig. 3).

##### Steroid type subgroup

**Fig. 4:**
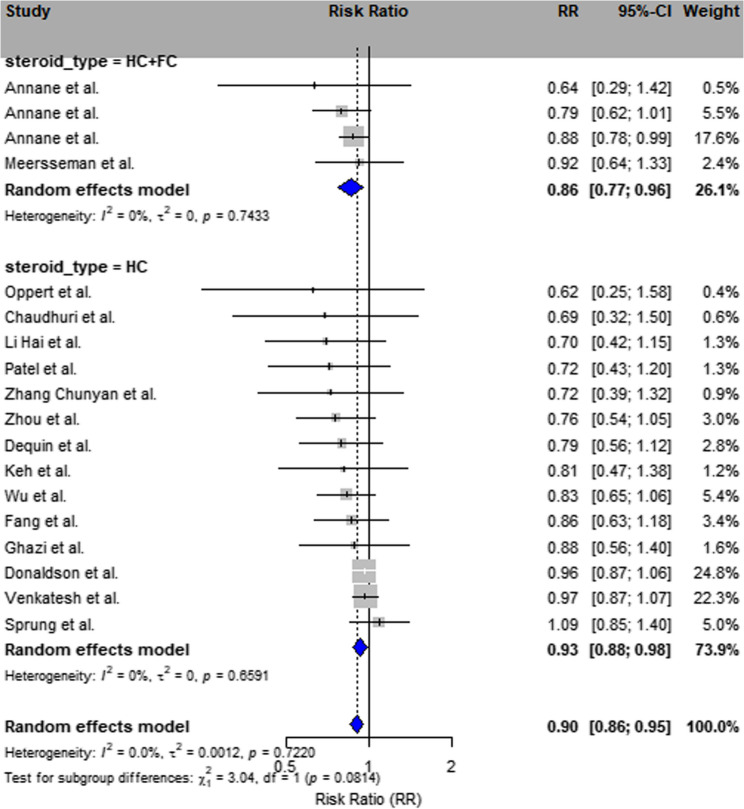
Subgroup analysis by steroid type Forest plot stratified by type of corticosteroid regimen: hydrocortisone alone (10 studies), hydrocortisone plus fludrocortisone (2 studies), and other glucocorticoids such as methylprednisolone or dexamethasone (6 studies). Note: For visual clarity, heterogeneity indicators were removed from the figure. Refer to the supplementary tables for corresponding statistical metrics

Studies utilizing hydrocortisone combined with fludrocortisone (*n* = 5) consistently showed a reduction in mortality (RR = 0.89; 95% CI: 0.83–0.95; I² = 41%). - Studies employing hydrocortisone alone (*n* = 6) also demonstrated benefit (RR = 0.75; 95% CI: 0.62–0.91), albeit with higher heterogeneity (I² = 67%) (Insert Fig. 4).

##### Geographic subgroup analysis

**Fig. 5:**
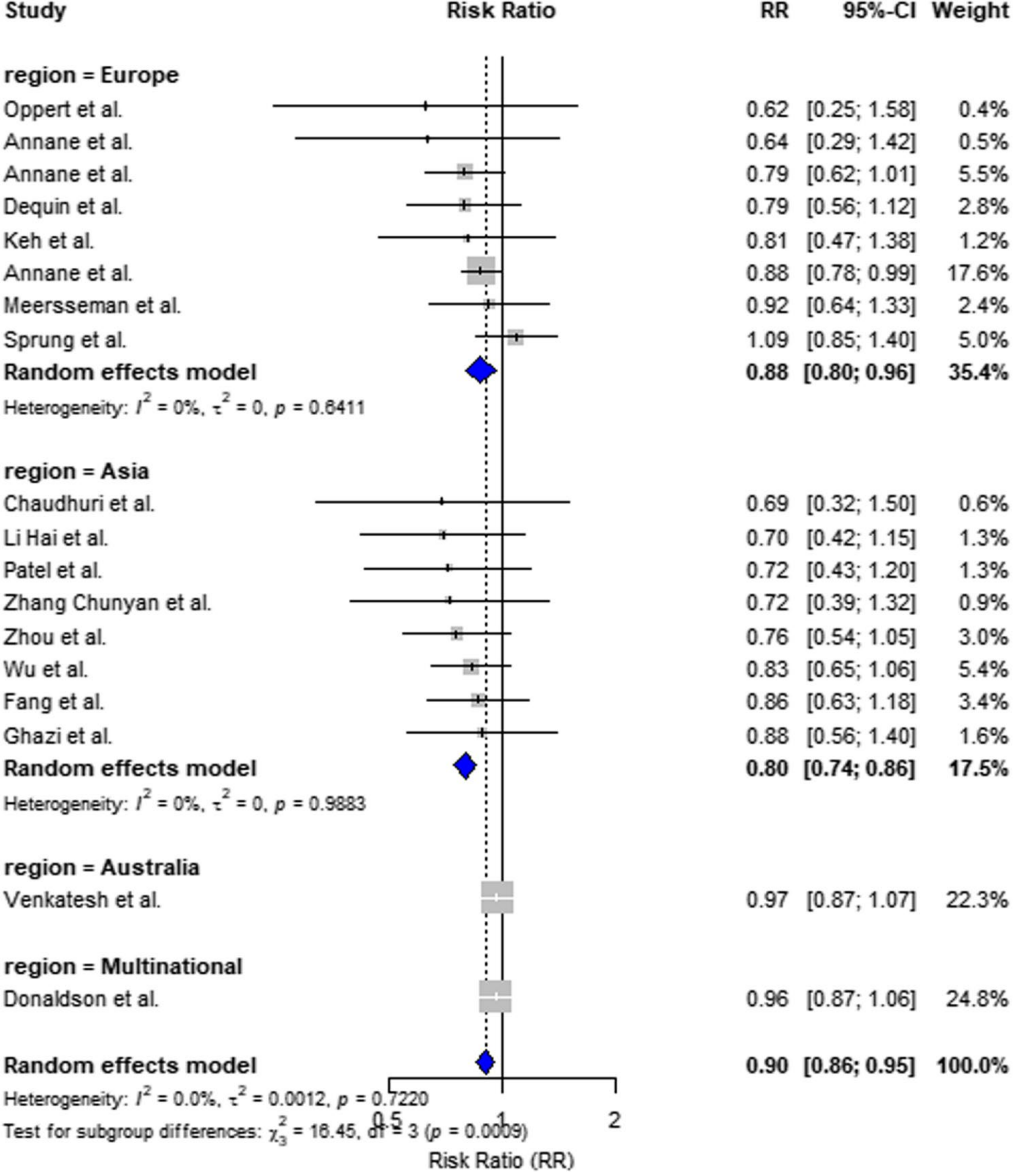
Subgroup analysis by study region Forest plot stratified by study region: China (6 studies) and non-China (12studies). Pooled RR and 95% CI are shown for each group. No significant heterogeneity was detected between regional subgroups (p for subgroup difference = 0.7556). Note: Heterogeneity statistics were excluded from this figure to avoid label overlapping. Full details are reported in the Results section and supplementary materials

In Chinese trials, the risk ratio (RR) was 0.93 (95% confidence interval [CI]: 0.87–1.00; I² = 0%), whereas in non-Chinese trials, the RR was 0.83 (95% CI: 0.74–0.92; I² = 63.8%) (Insert Fig. 5).

An additional: post-hoc exploratory analysis was conducted based on the timing of corticosteroid initiation (early vs. late), as defined by each individual study. This analysis was not pre-specified in the original protocol but was included due to its potential clinical relevance.

Visual asymmetry was evident on the funnel plot (see Supplementary Figure S3), and Egger’s test indicated small-study effects (t = − 4.01, *p* = 0.001; Supplementary Figure S4). A trim-and-fill analysis showed a slightly attenuated yet directionally consistent effect (Supplementary Figure S5).

Furthermore, Egger’s regression test demonstrated statistically significant evidence of publication bias (t = − 4.01, *p* = 0.001), consistent with the asymmetry observed in the funnel plot (Supplementary Figure S3). A trim-and-fill analysis (Supplementary Figure S5) showed slightly attenuated but directionally consistent results.

## Discussion

In this updated meta-analysis of 18 randomized controlled trials comprising 7,982 patients, corticosteroid therapy was associated with a significant reduction in 28-day mortality (RR = 0.88, 95% CI: 0.79–0.98), with moderate heterogeneity (I² = 39%). Subgroup analyses revealed that the most pronounced benefit occurred with moderate-dose corticosteroids (201–300 mg/day; RR = 0.86, I² = 0%) and with hydrocortisone plus fludrocortisone combination therapy (RR = 0.89). Regional analyses showed consistent but less pronounced effects in Chinese trials (RR = 0.93), compared to non-Chinese trials (RR = 0.83). The GRADE assessment rated the overall certainty of evidence for 28-day mortality as high.

Utilizing the GRADE approach, the overall certainty of evidence for the primary outcome of 28-day mortality was assessed as high, whereas the evidence for 90-day mortality and adverse events was rated as moderate and low, respectively. These findings suggest a substantial but time-limited benefit of corticosteroid treatment, warranting caution regarding long-term outcomes and safety.

Our study builds upon and extends the findings of previous meta-analyses, notably the Cochrane review by Annane et al. (2019), which assessed 61 trials. The distinct contribution of our analysis is the inclusion of ten trials conducted after 2020, a stratified dose-response assessment, and comparative geographic analysis. These enhancements address unresolved issues from earlier syntheses, which often combined heterogeneous dosing regimens or lacked geographic diversity.

Our study adds important granularity to the current literature by providing a stratified meta-analysis of corticosteroid dosing in sepsis. Specifically, we identified the 201–300 mg/day range as the most effective dosing window, with the lowest heterogeneity (I² = 0%) and statistically significant mortality reduction (RR = 0.86, 95% CI: 0.81–0.92).A recent large-scale pairwise and dose-response meta-analysis of 45 RCTs (*n* ≈ 9,500) published in Critical Care Explorations identified an optimal hydrocortisone equivalent dose of ~ 260 mg/day, while also reporting increased risks of hyperglycemia, hypernatremia, and neuromuscular weakness with higher dosing [27].While prior reviews have broadly supported low-to-moderate dosing, they lacked precise stratification. While prior reviews have broadly supported low-to-moderate dosing, they lacked precise stratification. For instance, Liang et al. (2021) conducted a systematic review of 50 RCTs and reported that low-to-moderate doses (≤ 200 mg/day hydrocortisone equivalent) yielded more stable benefits, whereas higher doses conferred no additional survival advantage and were associated with increased adverse events [28]. Our analysis extends these findings by formally comparing adjacent dose strata (≤ 200 mg, 201–300 mg, and >300 mg) and identifying 201–300 mg/day as.

range with the lowest heterogeneity.Our stratified subgroup analysis complements prior dose–response evaluations, such as the Bayesian meta-analysis by Son et al. (2021), which supported low-to-moderate dosing but did not delineate narrower windows (e.g., 200–300 mg/day) with the same level of precision [8].For instance, the Bayesian meta-regression by Moran et al. suggested ~ 260 mg/day as optimal, but did not formally compare adjacent dose ranges [29]. The network meta-analysis published in Critical Care Explorations proposed 200–400 mg/day as a likely effective range, yet grouped a wide range of regimens together without discrete subgroup analysis [30]. The Frontiers in Immunology review concluded that higher doses offer no additional benefit and may pose safety concerns, but again lacked stratified dosing data [28].In contrast, our findings provide direct, dose-specific comparative evidence across low, moderate, and high dosing categories, offering the most specific empirical support to date for selecting moderate-dose corticosteroids (201–300 mg/day) in sepsis management.

In our steroid-type subgroup analysis, both hydrocortisone monotherapy and combination therapy with fludrocortisone were associated with mortality reduction. Specifically, combination therapy (RR = 0.89; 95% CI: 0.83–0.95; I² = 41%) showed more consistent benefit across studies, while hydrocortisone alone (RR = 0.75; 95% CI: 0.62–0.91) was associated with greater heterogeneity (I² = 67%).

These findings are consistent with previous reports, including a large retrospective cohort that demonstrated a significant mortality benefit of fludrocortisone plus hydrocortisone, and an individual patient-level meta-analysis supporting the potential role of combination therapy in septic shock [5, 31]While our analysis does not directly compare monotherapy versus combination therapy within individual trials, it provides stratified insights into the consistency of treatment effects. The lower heterogeneity observed in the fludrocortisone subgroup suggests a more uniform benefit, warranting further prospective evaluation of its additive value.A recent randomized dose–response trial (FluDReSS) further examined the adjunctive use of enteral fludrocortisone in septic shock, comparing hydrocortisone alone with three fludrocortisone doses (50 µg, 100 µg, and 200 µg daily) [32]. Although time to shock resolution did not differ significantly among groups, the highest-dose arm (200 µg) demonstrated a lower observed 28-day mortality (≈ 11% vs. 24% with hydrocortisone monotherapy) and favorable safety profile, while pharmacokinetic analyses revealed substantial inter-individual variability in absorption [32]. These findings complement our subgroup analysis by reinforcing the potential additive benefit of fludrocortisone, while underscoring the importance of optimized dosing and the need for further pharmacokinetic-guided trials.

Our analysis identified a distinct regional disparity in corticosteroid efficacy. Specifically, Chinese trials demonstrated a modest but consistent benefit (RR = 0.93; I² = 0%), whereas non-Chinese trials exhibited a more pronounced effect (RR = 0.83) but with substantial heterogeneity (I² = 63.8%). This contrast suggests that while corticosteroids may appear more effective in certain non-Chinese settings, the variability in outcomes is greater—possibly due to differences in ICU protocols, trial conduct, patient selection, and corticosteroid dosing strategies.Notably, all Chinese trials employed hydrocortisone monotherapy at relatively low doses, which may partially account for the attenuated but consistent treatment effects observed. This pattern highlights an important nuance: although more pronounced effects were observed in some non-Chinese studies, their stability was lower. In contrast, the Chinese trials—despite showing weaker effect sizes—yielded highly consistent results. This tradeoff between magnitude and consistency underscores the potential influence of region-specific treatment practices on outcome heterogeneity.While previous reviews and guidelines have acknowledged global variability in clinical practice, the impact of these contextual factors on effect estimates has rarely been formally quantified. Angus and van der Poll described major cross-national differences in sepsis management, particularly in vasopressor timing and supportive care intensity, but these insights were largely narrative [33]. Similarly, the APROCCHSS trial by Annane et al. demonstrated significant benefit from fludrocortisone–hydrocortisone combination therapy, but its findings are based entirely on standardized European settings [4].Moreover, current sepsis guidelines—such as those by Lamontagne et al.—draw heavily on Western data while acknowledging the lack of robust RCTs from Asia [34]. By stratifying trials by geographic region, our study helps fill this gap and offers empirical support for tailoring recommendations to regional treatment realities. The observed divergence reinforces the need for multicenter, high-quality RCTs in Asia to better define optimal steroid strategies across diverse healthcare settings.

Timing of corticosteroid initiation and baseline disease severity may also modulate treatment efficacy.For instance, in patients with COVID-19, delayed initiation of corticosteroids has been associated with attenuated therapeutic benefit, especially among critically ill individuals [35].These findings suggest that early administration may be crucial in maximizing the anti-inflammatory effects of corticosteroids.While sepsis and COVID-19 differ in pathophysiology, both syndromes involve dysregulated host responses and a narrow therapeutic window for immunomodulation.Accordingly, future sepsis trials should consider stratifying patients not only by dose and steroid type, but also by timing and baseline severity to refine clinical recommendations.

Despite the consistent benefit observed for the primary endpoint of 28-day mortality, several limitations warrant attention. Notably, long-term outcomes—such as 90-day or 6-month mortality—were infrequently reported, and adverse events like hyperglycemia, secondary infections, or myopathy were inconsistently documented across studies. This aligns with a recent systematic review that quantified increased hyperglycemia risk while confirming limited evidence for other adverse outcomes [36].As such, the safety profile of corticosteroid therapy in sepsis remains incompletely characterized [36]. Future meta-analyses incorporating individual patient data (IPD) are needed to better quantify these outcomes and evaluate risk-benefit trade-offs with greater granularity.

Accordingly, future randomized trials should consider stratifying patients not only by dosage and corticosteroid type, but also by disease severity and treatment timing to refine clinical recommendations.Finally, although we applied a rigorous inclusion process, several well-known randomized trials were excluded due to discrepancies in population, intervention strategies, or incomplete outcome reporting. To ensure transparency, these studies and the reasons for their exclusion are summarized in Supplementary Table S5. In addition, the inconsistent diagnostic definitions of sepsis and septic shock used across trials may contribute to underlying clinical heterogeneity. These definitions have been compiled in Supplementary Table S3 to improve interpretability and facilitate future harmonization.Moreover, given the persistent paucity of high-quality data from Asia and other underrepresented regions, future research should prioritize large-scale, multicenter randomized controlled trials in these settings. Additionally, individual patient data meta-analyses (IPD-MAs) are warranted to better characterize treatment-response heterogeneity and optimize patient-specific corticosteroid strategies.These recommendations are consistent with recent expert reviews emphasizing the evolution of corticosteroid strategies in septic shock, advocating for moderate dosing, individualized treatment plans, and context-specific implementation based on local healthcare realities [37].

Publication bias remains a potential concern in meta-analyses, particularly when recent trials show smaller or null effects. The Egger’s regression test revealed statistically significant evidence of publication bias (t = −4.01, *p* = 0.001), consistent with the asymmetry observed in the funnel plot (Supplementary Figure S3). To further assess the potential impact of missing studies, we performed a trim-and-fill analysis (Supplementary Figure S4), which estimated the adjusted pooled effect size after accounting for unpublished trials. Although the imputed effect was slightly attenuated, the directionality of benefit remained unchanged, reinforcing the robustness of our findings.

## Conclusion

This updated meta-analysis demonstrates that low-to-moderate dose corticosteroids—specifically intravenous hydrocortisone at 200–300 mg/day—are associated with a significant reduction in 28-day mortality among adult patients with sepsis or septic shock. These findings are consistent with current guideline recommendations and support the integration of corticosteroids as part of standard supportive care.

By incorporating dose-stratified and region-specific subgroup analyses, our study adds clarity to prior heterogeneous syntheses and highlights the importance of tailoring steroid regimens based on patient characteristics, institutional resources, and geographic context.These findings align with the 2021 Surviving Sepsis Campaign, which conditionally recommends hydrocortisone at 200 mg/day for refractory septic shock despite low-to-moderate certainty (SSC, 2021) [3]. Notably, the consistent but modest effects observed in Chinese trials contrast with the greater yet more variable effects in non-Chinese studies, emphasizing the need for regionally adapted treatment strategies.

However, long-term outcomes (e.g., 90-day mortality) and safety profiles—particularly concerning hyperglycemia, infection risk, and myopathy—remain insufficiently reported in the existing literature. Future randomized trials should evaluate these endpoints and consider stratifying patients by dose, steroid type, timing of administration, and baseline severity to better personalize treatment.

Although this meta-analysis was not prospectively registered on PROSPERO or any public platform due to its retrospective design and reliance on previously published data, a predefined protocol was developed and rigorously followed. Nevertheless, the lack of formal registration is acknowledged as a methodological limitation.

In conclusion, moderate-dose corticosteroids—with or without adjunctive fludrocortisone—represent a rational, evidence-based adjunct in the management of septic shock, pending further confirmation from large-scale, multicenter trials.

## Supplementary Information

Supplementary Figure S1. PRISMA flowchart. Records identified from PubMed, Embase, CENTRAL, Web of Science, SCOPUS, ClinicalTrials.gov (n = 3,120); after duplicates removed n = 2,013; full‑text assessed n = 68; RCTs included n = 18.

Supplementary Figure S2. Leave-one-out sensitivity analysis. Pooled risk ratios (RR) recalculated after exclusion of each individual study. The dashed red line indicates the overall effect estimate. Consistency of the results confirms the robustness of the findings.

Supplementary Figure S3. Funnel plot. The funnel plot indicates asymmetry among the included studies. Egger’s regression test demonstrated statistically significant publication bias (t = –4.01, p = 0.001).

Supplementary Figure S4. Egger’s regression test plot. Scatter plot of standard error vs. effect size (precision). The significant intercept (t = –4.01, p = 0.001) suggests presence of publication bias.

Supplementary Figure S5. Trim-and-fill analysis. The trim-and-fill funnel plot displays observed studies (black circles) and imputed studies (gray triangles). The adjusted pooled effect was slightly reduced but maintained directional consistency, suggesting robustness of findings despite potential bias.

Supplementary Table S1: Study Protocol. Note: Provides the predefined study protocol outlining eligibility criteria, search strategy, and planned subgroup analyses.

Supplementary Table S2: PICOS Framework Criteria. Note: Summarizes population, intervention, comparator, outcomes, and study design (PICOS) used to define inclusion/exclusion criteria.

Supplementary Table S3: Sepsis and Septic Shock Diagnostic Definitions. Note: Lists the diagnostic criteria (Sepsis-3, ACCP/SCCM, or study-defined) applied in each included trial.

Supplementary Table S4: Outcome Reporting (28-day, 90-day, Adverse Events). Note: Details which studies reported short-term mortality, long-term mortality, and adverse events.

Supplementary Table S5: Excluded Randomized Controlled Trials and Reasons. Note: Lists major RCTs excluded during full-text screening, with specific reasons for exclusion.

Supplementary Table S6: Study-level Data for Subgroup Analyses. Note: Provides trial-level characteristics and effect estimates used in subgroup analyses (dose, region, steroid type).

## Data Availability

The datasets used and/or analyzed during the current study are available from the corresponding author on reasonable request.
