# Supplementary figures and images for: Corticosteroids for sepsis and septic shock: a meta-analysis of 18 RCTs with dose-stratified and fludrocortisone subgroup evaluation
Source: BMC Anesthesiol. 2025 Oct 21;25:511. doi: 10.1186/s12871-025-03388-1 (PMC12538775; doi:10.1186/s12871-025-03388-1)

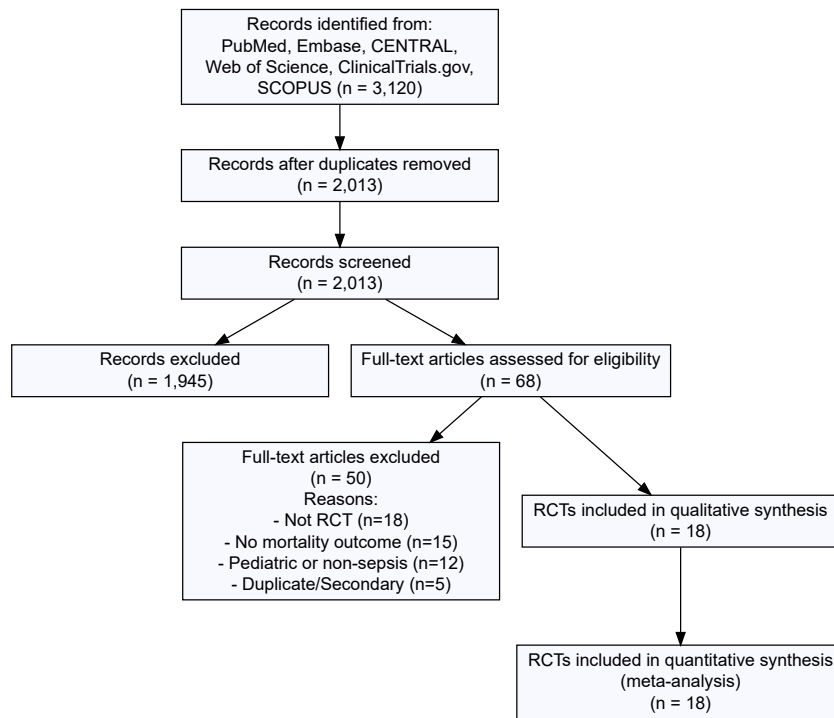

Supplement: Supplementary file 1 — Supplementary Figure S1. PRISMA flowchart. Records identified from PubMed, Embase, CENTRAL, Web of Science, SCOPUS, ClinicalTrials.gov (n = 3,120); after duplicates removed n = 2,013; full‑text assessed n = 68; RCTs included n = 18. [file 12871_2025_3388_MOESM1_ESM.pdf]

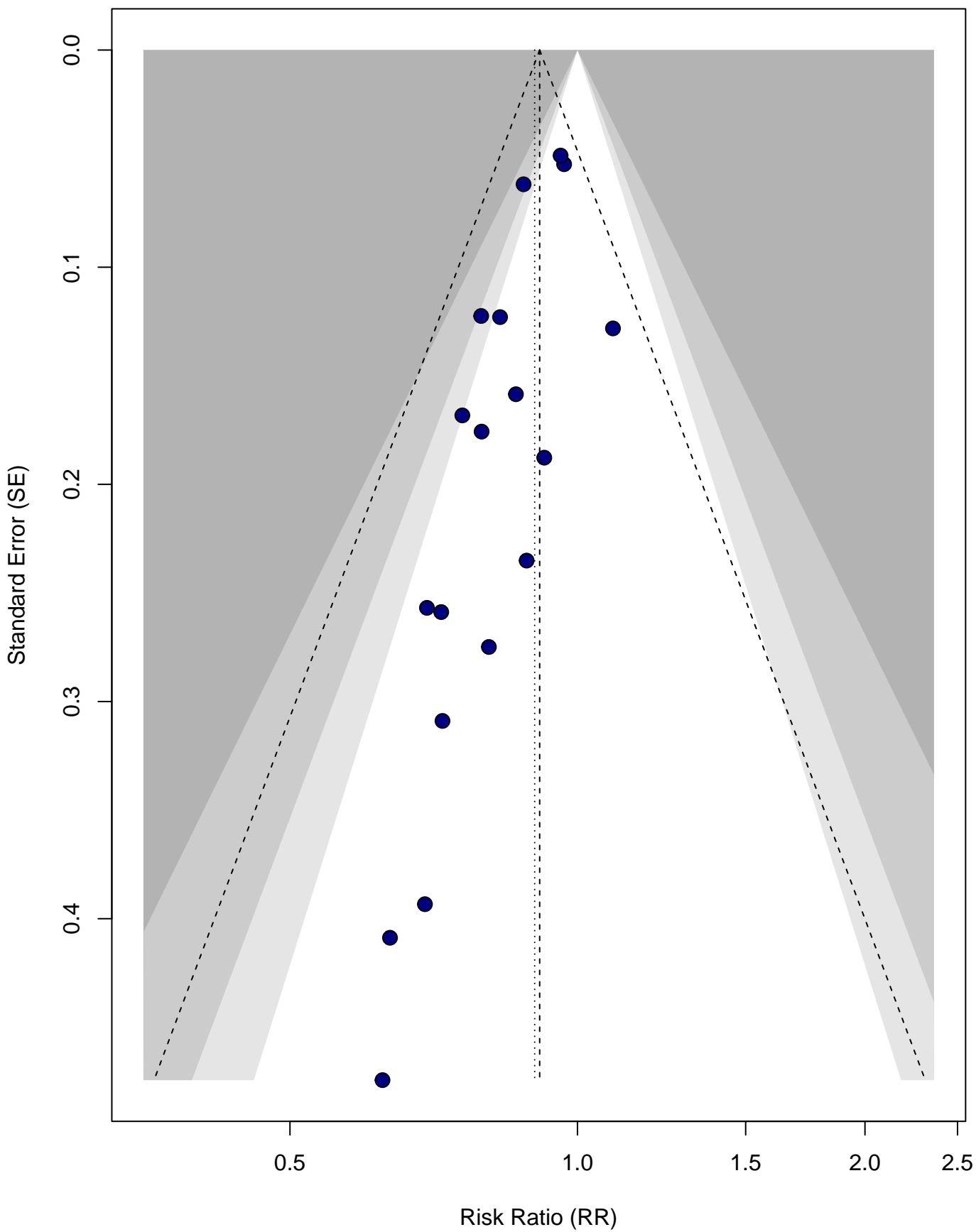

Supplement: Supplementary file 3 — Supplementary Figure S3. Funnel plot. The funnel plot indicates asymmetry among the included studies. Egger’s regression test demonstrated statistically significant publication bias (t = –4.01, p = 0.001). [file 12871_2025_3388_MOESM3_ESM.pdf]

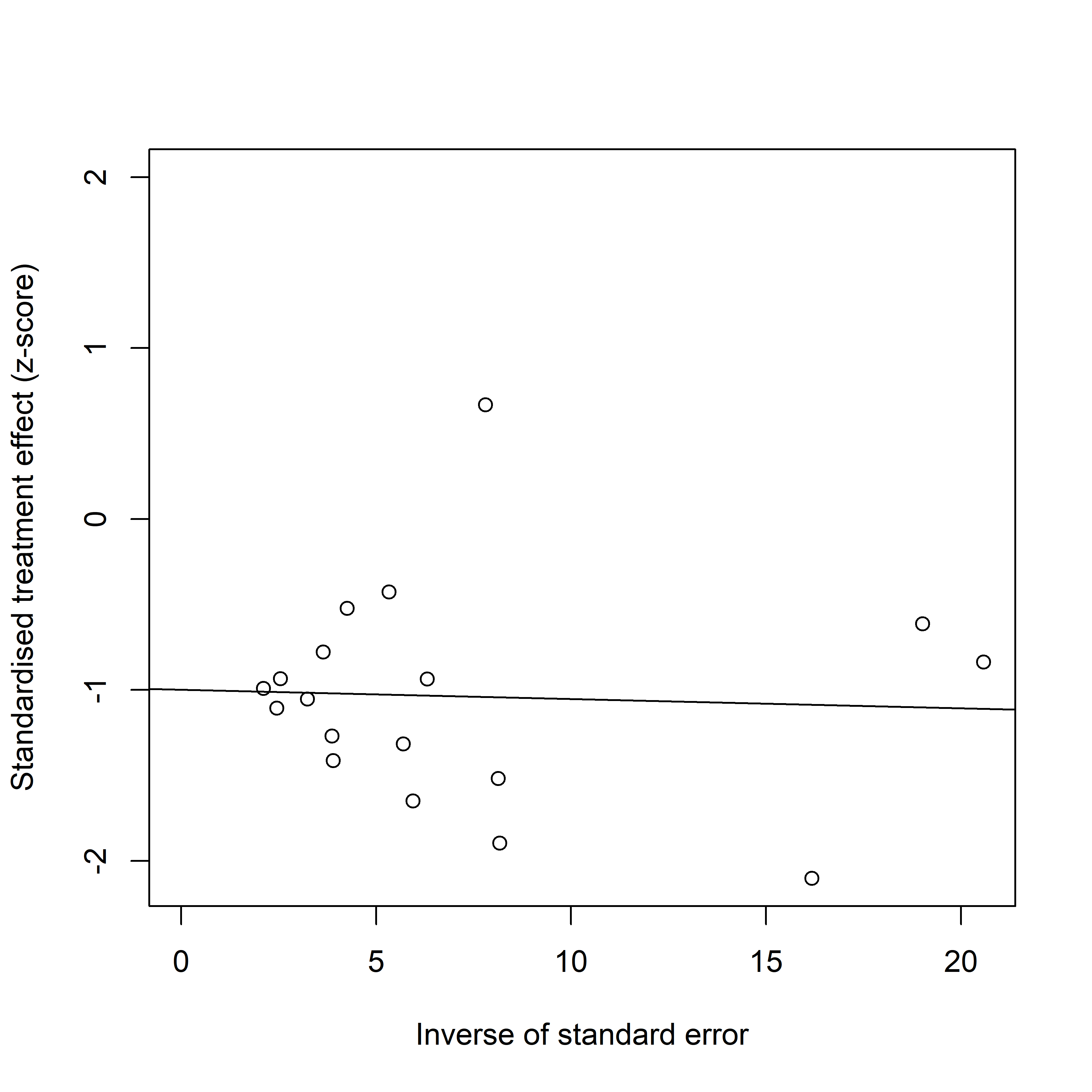

Supplement: Supplementary file 4 — Supplementary Figure S4. Egger’s regression test plot. Scatter plot of standard error vs. effect size (precision). The significant intercept (t = –4.01, p = 0.001) suggests presence of publication bias. [file 12871_2025_3388_MOESM4_ESM.tiff]

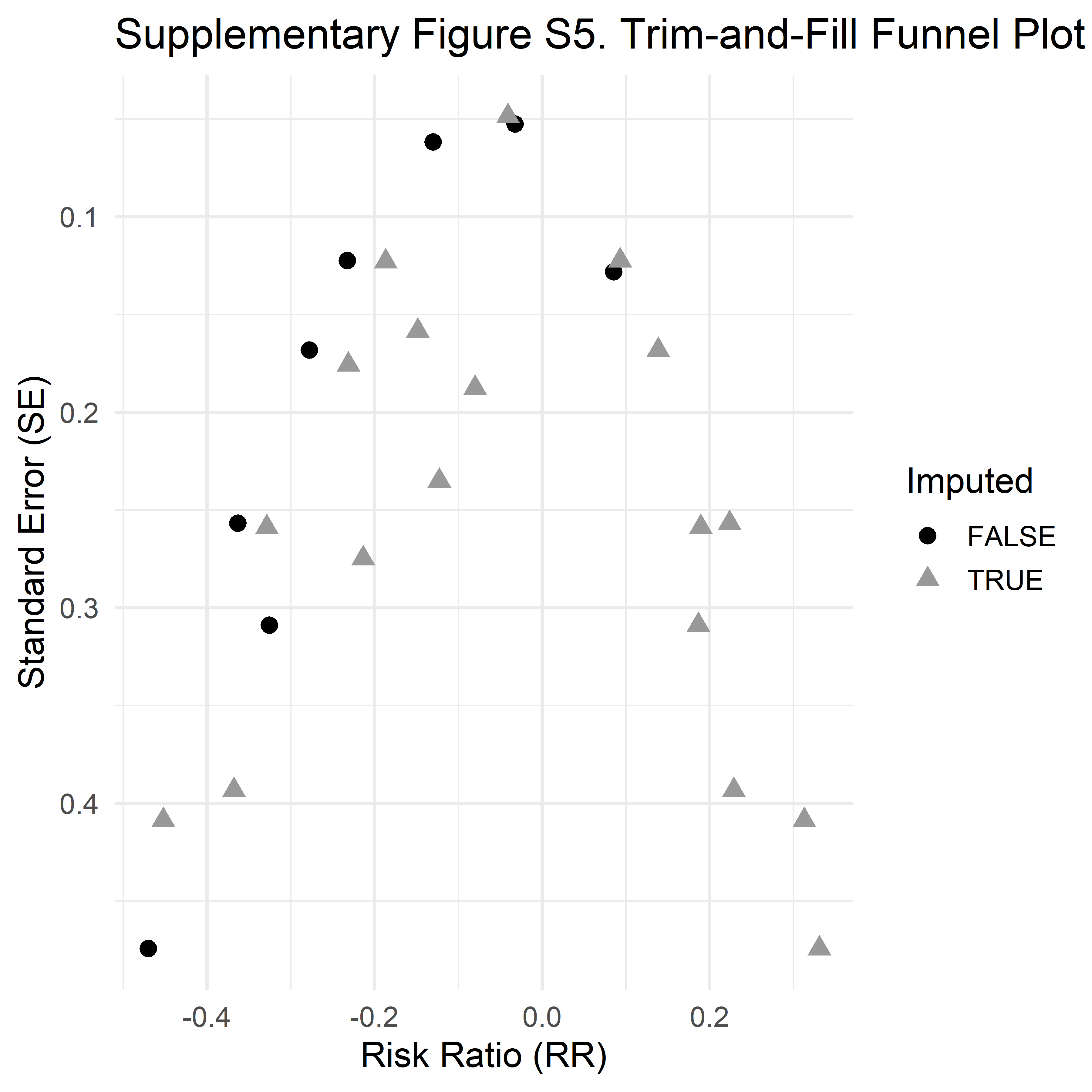

Supplement: Supplementary file 5 — Supplementary Figure S5. Trim-and-fill analysis. The trim-and-fill funnel plot displays observed studies (black circles) and imputed studies (gray triangles). The adjusted pooled effect was slightly reduced but maintained directional consistency, suggesting robustness of findings despite potential bias. [file 12871_2025_3388_MOESM5_ESM.tiff]
