# Supplementary material for: Corticosteroids for sepsis and septic shock: a meta-analysis of 18 RCTs with dose-stratified and fludrocortisone subgroup evaluation
Source: BMC Anesthesiol. 2025 Oct 21;25:511. doi: 10.1186/s12871-025-03388-1 (PMC12538775; doi:10.1186/s12871-025-03388-1)
Supplement: Supplementary file 6 — Supplementary Table S1: Study Protocol. Note: Provides the predefined study protocol outlining eligibility criteria, search strategy, and planned subgroup analyses. [file 12871_2025_3388_MOESM6_ESM.docx]

# Protocol for Study Selection and Analysis

Title: Protocol for Study Selection and Data Analysis in the Meta-analysis of Corticosteroids for Sepsis and Septic Shock

## **1. Objective**

To evaluate the effect of corticosteroids on 28-day mortality in adult patients with sepsis and septic shock, using dose-stratified and fludrocortisone subgroup analysis based on randomized controlled trials (RCTs).

## **2. Eligibility Criteria**

1. Inclusion:
2. Study design: Randomized controlled trials (RCTs)
3. Population: Adults (≥18 years) with sepsis or septic shock
4. Intervention: Corticosteroids (hydrocortisone, dexamethasone, methylprednisolone, etc.)
5. Comparator: Placebo or standard care
6. Outcomes: At least one of the following—28-day mortality (primary), 90-day mortality, or adverse events
7. Language: No restrictions. Non‑English studies were eligible if full texts were accessible and eligibility criteria were met.
8. Exclusion:
9. Non-RCTs, observational studies, animal studies
10. Pediatric populations
11. Trials without mortality outcomes or with insufficient data for effect size estimation

## **3. Search Strategy**

Databases searched:

1. PubMed
2. Embase
3. Cochrane CENTRAL
4. Web of Science
5. ClinicalTrials.gov

Time frame: Inception to April 1, 2024

Search terms included combinations of:

“sepsis” OR “septic shock”

AND “corticosteroids” OR “hydrocortisone” OR “dexamethasone” OR “fludrocortisone”

Reference lists of relevant reviews and articles were also screened manually.

## **4. Study Selection Process**

1. Two reviewers independently screened titles and abstracts.
2. Full texts of potentially relevant articles were assessed using predefined criteria.
3. Discrepancies were resolved by consensus or by a third reviewer.

A PRISMA 2020-compliant flow diagram was constructed to illustrate the selection process (Figure 1 in main text).

## **5. Data Extraction**

Extracted data included:

1. Author, year, sample size
2. Study region
3. Intervention details (steroid type, dose, duration)
4. Outcome measures (28-day mortality, 90-day mortality, adverse events)
5. Randomization, blinding, and allocation concealment details

Data were extracted using a standardized Excel template by two reviewers independently.

## **6. Risk of Bias Assessment**

Each included study was evaluated using the Cochrane Risk of Bias 2.0 tool, assessing five domains:

1. Randomization process
2. Deviations from intended interventions
3. Missing outcome data
4. Outcome measurement
5. Selection of the reported result

Disagreements were resolved through discussion.

## **7. Statistical Analysis**

1. Pooled risk ratios (RRs) and 95% confidence intervals (CIs) were calculated.
2. A random-effects model (DerSimonian–Laird) was used for primary analysis.
3. Subgroup analyses were pre-specified for:
4. Corticosteroid dose (≤200, 201–300, >300 mg/day hydrocortisone-equivalent)
5. Steroid type (hydrocortisone alone vs. hydrocortisone + fludrocortisone)
6. Region (China vs. non-China)
7. Heterogeneity was assessed via I² statistic.
8. Publication bias was evaluated by funnel plot and Egger’s test.
9. Sensitivity analysis included leave-one-out approach.

Software used: RevMan 5.4, R (meta and metafor packages)

## **8. Registration**

This study was not registered on PROSPERO or other registries, but the protocol described herein was finalized before literature screening and data extraction began.
