# Supplementary material for: Corticosteroids for sepsis and septic shock: a meta-analysis of 18 RCTs with dose-stratified and fludrocortisone subgroup evaluation
Source: BMC Anesthesiol. 2025 Oct 21;25:511. doi: 10.1186/s12871-025-03388-1 (PMC12538775; doi:10.1186/s12871-025-03388-1)
Supplement: Supplementary file 7 — Supplementary Table S2: PICOS Framework Criteria. Note: Summarizes population, intervention, comparator, outcomes, and study design (PICOS) used to define inclusion/exclusion criteria. [file 12871_2025_3388_MOESM7_ESM.docx]

# Supplementary Table 2. PICOS Criteria Used for Study Inclusion in This Meta-analysis

| Component | Description |
| --- | --- |
| Population (P) | Adults (≥18 years) diagnosed with sepsis or septic shock according to definitions provided in the original trials (e.g., ACCP/SCCM, Sepsis-3). |
| Intervention (I) | Systemic corticosteroid therapy, including hydrocortisone, dexamethasone, methylprednisolone, or prednisone, with or without fludrocortisone. |
| Comparison (C) | Placebo or standard care without corticosteroids. |
| Outcomes (O) | Primary: 28-day mortality. Secondary: 90-day mortality, adverse events (e.g., GI bleeding, secondary infections, hyperglycemia). |
| Study Design (S) | Randomized Controlled Trials (RCTs), with no language restrictions. Non-English studies were included if full texts were accessible and eligibility criteria were met. |
