# Supplementary material for: Corticosteroids for sepsis and septic shock: a meta-analysis of 18 RCTs with dose-stratified and fludrocortisone subgroup evaluation
Source: BMC Anesthesiol. 2025 Oct 21;25:511. doi: 10.1186/s12871-025-03388-1 (PMC12538775; doi:10.1186/s12871-025-03388-1)
Supplement: Supplementary file 8 — Supplementary Table S3: Sepsis and Septic Shock Diagnostic Definitions. Note: Lists the diagnostic criteria (Sepsis-3, ACCP/SCCM, or study-defined) applied in each included trial. [file 12871_2025_3388_MOESM8_ESM.docx]

# Supplementary Table S3. Definitions of Sepsis and Septic Shock in Included RCTs

| Study | Year | Definition of Sepsis/Septic Shock |
| --- | --- | --- |
| Briegel et al. | 1999 | Sepsis defined by ACCP/SCCM criteria; septic shock as hypotension despite fluid resuscitation. |
| Annane et al. | 2002 | Sepsis per ACCP/SCCM; septic shock requiring vasopressors and mechanical ventilation. |
| Gibot et al. | 2006 | Sepsis with confirmed infection and organ dysfunction; shock defined as persistent hypotension. |
| Carenzi et al. | 2009 | Sepsis per national criteria; shock defined as MAP <65 mmHg despite fluids. |
| Sprung et al. | 2008 | Septic shock defined as persistent hypotension with vasopressor requirement and hypoperfusion. |
| Annane et al. | 2010 | Sepsis per ACCP/SCCM; septic shock defined as vasopressor need >1 hr despite fluids. |
| Liu et al. | 2012 | Chinese guidelines: sepsis defined by infection and SIRS; shock as unresponsive hypotension. |
| Tong et al. | 2013 | Diagnosis based on infection plus organ dysfunction; shock defined by SBP <90 mmHg after fluids. |
| Du et al. | 2014 | Sepsis diagnosed with suspected infection, elevated SOFA; shock defined as lactate >2 mmol/L + vasopressors. |
| Tong et al. | 2015 | Same center criteria; septic shock defined by SBP <90 mmHg requiring vasopressors. |
| Xie et al. | 2017 | Chinese guidelines; shock defined as hypotension not corrected by fluid resuscitation. |
| Venkatesh et al. | 2018 | Sepsis-3; septic shock as persistent hypotension requiring vasopressors + lactate >2 mmol/L. |
| Cui et al. | 2019 | Sepsis with infection and organ failure; shock as sustained hypotension despite fluids. |
| Xie et al. | 2021 | Sepsis per Sepsis-3; shock defined by vasopressor use + lactate >2 mmol/L. |
| Zhou et al. | 2022 | Infection + organ dysfunction; shock defined by norepinephrine support >1 hour. |
| Xiao et al. | 2023 | Sepsis defined by SOFA ≥2; shock as MAP <65 despite adequate fluids. |
| Tong et al. | 2023 | Diagnosis by Sepsis-3; shock defined as hypotension + lactate >2. |
| Zhang et al. | 2023 | Sepsis and shock defined using Sepsis-3 and Chinese critical care consensus. |
