# Supplementary material for: Corticosteroids for sepsis and septic shock: a meta-analysis of 18 RCTs with dose-stratified and fludrocortisone subgroup evaluation
Source: BMC Anesthesiol. 2025 Oct 21;25:511. doi: 10.1186/s12871-025-03388-1 (PMC12538775; doi:10.1186/s12871-025-03388-1)
Supplement: Supplementary file 10 — Supplementary Table S5: Excluded Randomized Controlled Trials and Reasons. Note: Lists major RCTs excluded during full-text screening, with specific reasons for exclusion. [file 12871_2025_3388_MOESM10_ESM.docx]

# Major Excluded Randomized Controlled Trials (RCTs) and Reasons for Exclusion

| Study (First Author, Year) | PMID | Country | Intervention | Reason for Exclusion |
| --- | --- | --- | --- | --- |
| Tongyoo, 2016 | 27837895 | Thailand | Hydrocortisone | Patient population included ARDS; sepsis not primary diagnosis |
| Jung, 2010 | 20042470 | South Korea | Dexamethasone | Used non-hydrocortisone corticosteroid (dexamethasone) |
| Leone, 2014 | 24486852 | France | Hydrocortisone | Reported only in-hospital mortality, not time-specific (e.g., 28-day) |
| Volbeda, 2018 | 29376852 | Netherlands | Hydrocortisone | Duplicate data from Sprung 2008 (already included) |
| Annane, 2016 | 26903338 | France | Hydrocortisone + Fludrocortisone | Review article; not a primary RCT |
| Arabi, 2021 | 33404146 | Saudi Arabia | Hydrocortisone in COVID-19 sepsis | COVID-19 specific; not general sepsis/septic shock |
